# Infectious Diseases and Basal Ganglia Calcifications: A Cross-Sectional Study in Patients with Fahr’s Disease and Systematic Review

**DOI:** 10.3390/jcm13082365

**Published:** 2024-04-18

**Authors:** Birgitta M. G. Snijders, Mike J. L. Peters, Susanne van den Brink, Marijke J. C. A. van Trijp, Pim A. de Jong, Laurens A. T. M. Vissers, Frans M. Verduyn Lunel, Marielle H. Emmelot-Vonk, Huiberdina L. Koek

**Affiliations:** 1 Department of Geriatrics, University Medical Center Utrecht, 3584 CX Utrecht, The Netherlands; 2 Department of Internal Medicine, University Medical Center Utrecht, 3584 CX Utrecht, The Netherlands; 3 Department of Geriatrics, ZorgSaam Hospital, 4535 PA Terneuzen, The Netherlands; 4 Department of Microbiology, Groene Hart Ziekenhuis, 2803 HH Gouda, The Netherlands; 5 Department of Radiology, University Medical Center Utrecht, 3584 CX Utrecht, The Netherlands; 6 Department of Medical Microbiology, University Medical Center Utrecht, 3584 CX Utrecht, The Netherlands

**Keywords:** basal ganglia, calcification, infection, Fahr’s disease, primary familial brain calcification, systematic review

## Abstract

**Background:** It is unclear whether patients with basal ganglia calcifications (BGC) should undergo infectious disease testing as part of their diagnostic work-up. We investigated the occurrence of possibly associated infections in patients with BGC diagnosed with Fahr’s disease or syndrome and consecutively performed a systematic review of published infectious diseases associated with BGC. **Methods:** In a cross-sectional study, we evaluated infections in non-immunocompromised patients aged ≥ 18 years with BGC in the Netherlands, who were diagnosed with Fahr’s disease or syndrome after an extensive multidisciplinary diagnostic work-up. Pathogens that were assessed included the following: *Brucella* sp., cytomegalovirus, human herpesvirus type 6/8, human immunodeficiency virus (HIV), *Mycobacterium tuberculosis*, rubella virus, and *Toxoplasma gondii*. Next, a systematic review was performed using MEDLINE and Embase (2002–2023). **Results:** The cross-sectional study included 54 patients (median age 65 years). We did not observe any possible related infections to the BGC in this population. Prior infection with *Toxoplasma gondii* occurred in 28%, and in 94%, IgG rubella antibodies were present. The positive tests were considered to be incidental findings by the multidisciplinary team since these infections are only associated with BGC when congenitally contracted and all patients presented with adult-onset symptoms. The systematic search yielded 47 articles, including 24 narrative reviews/textbooks and 23 original studies (11 case series, 6 cross-sectional and 4 cohort studies, and 2 systematic reviews). Most studies reported congenital infections associated with BGC (cytomegalovirus, HIV, rubella virus, Zika virus). Only two studies reported acquired pathogens (chronic active Epstein–Barr virus and *Mycobacterium tuberculosis*). The quality of evidence was low. **Conclusions:** In our cross-sectional study and systematic review, we found no convincing evidence that acquired infections are causing BGC in adults. Therefore, we argue against routine testing for infections in non-immunocompromised adults with BGC in Western countries.

## 1. Introduction

Basal ganglia calcifications (BGC) are a common radiological finding with an estimated prevalence of 1.3% in the general population [1]. BGC can develop as a consequence of a genetic disease, endocrine disorders, intoxications, the natural aging process, or infections [2]. Fahr’s disease, also known as Primary Familial Brain Calcification (PFBC), is an example of a rare neurodegenerative hereditary disease that is characterized by bilateral symmetrical BGC [3]. Thus far, seven pathogenic genetic mutations have been identified associated with PFBC [4]. The term Fahr’s syndrome is often used when BGC are secondary to another cause as aforementioned [2]. Identifying the etiology of BGC is important as treatment of the underlying cause might improve symptoms or even resolve calcifications, for example, in congenital infections with *Toxoplasma gondii* [5]. Therefore, as the diagnosis may have implications for the treatment regimen and prognosis, an extensive diagnostic work-up is nowadays recommended in patients with BGC, including testing for infections [2,3,6].

Several infectious diseases, both congenital and acquired, have been associated with BGC. Congenital infections include the TORCH infections (an acronym which comprises the pathogens *Toxoplasma gondii*, rubella virus, cytomegalovirus (CMV), and herpes simplex virus (HSV)), and acquired infections include the pathogens *Brucella* sp., human immunodeficiency virus (HIV), and *Mycobacterium tuberculosis* [2]. The available evidence regarding infectious diseases associated with BGC is low as it consists solely of case reports, case series, scoping reviews, and textbooks. Since there is no consensus on which tests should be performed in patients with BGC, a broad panel of infectious disease diagnostics is often used in clinical practice [6,7]. Yet, it can be debated whether, for example, congenital infections must be ruled out in patients with adult-onset symptoms. The interpretation and relevance of positive results can be difficult to assess, while on the other hand, these outcomes can delay further diagnostic work-up due to alternative diagnostic considerations.

This study evaluated the association between infectious diseases and the presence of BGC in patients with BGC. First, we presented the findings of a pragmatic infectious disease diagnostic work-up in adult patients with Fahr’s disease or syndrome at our outpatient clinic. We investigated the occurrence of infections possibly associated with BCG in these patients. Next, we conducted a systematic review to explore which infectious diseases are associated with BGC in the literature. Based on these findings, we proposed evidence-based recommendations for the assessment of infectious diseases in the diagnostic work-up in adult patients with BGC.

## 2. Methods

### 2.1. Cross-Sectional Study

A cross-sectional study was performed at the University Medical Center Utrecht, Utrecht, the Netherlands. All patients aged ≥ 18 years who were suspected to have Fahr’s disease or syndrome and visited the outpatient clinic between 1 September 2019 and 1 June 2023 were eligible for inclusion. The University Medical Center Utrecht is an academic hospital in which most patients with (suspected) Fahr’s disease in the Netherlands are examined. Fahr’s disease and syndrome were diagnosed based on the presence of clinical symptoms consistent with a diagnosis of Fahr’s disease or syndrome and the presence of bilateral calcifications of the basal ganglia as seen on a Computed Tomography (CT) scan of the head, which were not (solely) due to the natural ageing process. The presence of a (likely) pathogenic variant in one of the PFBC-related genes and/or a positive family history for Fahr’s disease were supportive criteria for the diagnosis of Fahr’s disease, but not mandatory. If a secondary cause of BGC was identified, patients were diagnosed with Fahr’s syndrome. Patients who had neither a pathogenic mutation nor a secondary cause were classified as having Fahr’s disease. Patients were excluded from analysis when they were not diagnosed with Fahr’s disease or syndrome or when they did not give informed consent.

A multidisciplinary team was involved in the diagnostic assessment of each patient. All patients underwent a comprehensive diagnostic work-up during their first visit as part of standard care. The work-up consisted of obtaining medical history, medication review, physical examination, neuropsychological examination, comprehensive laboratory and microbiological testing, and neuroimaging with a brain CT and Magnetic Resonance Imaging (MRI) scan. If a CT or MRI scan had been recently performed in another hospital, the scan was not repeated at baseline. Therefore, scanning protocols differed per patient. The location and severity of intracranial calcification was assessed by a board-certified radiologist with a special interest and expertise in Fahr’s disease using the Total Calcification Score. The Total Calcification Score is a visual rating scale on CT scans that quantifies the presence of calcification in 18 different brain locations. Each location is attributed a score between 0 (absent calcification) and 5 (severe and confluent calcification) points. The Total Calcification Score is the sum of all score points and ranges from 0 to 90 [8]. Genetic testing was performed in patients who gave informed consent for this. The diagnostic procedures are described in more detail elsewhere [9].

An infectious disease diagnostic work-up was performed during the first visit as part of standard care. As we had not yet conducted this systematic review at the time of testing, nor have any other systematic review been published about this topic, a pragmatic approach was used to establish the infectious disease testing set. This set was based on expert opinion of the multidisciplinary team and a non-systematic literature search [2,10]. The work-up used peripheral blood samples and consisted of the following: *Brucella* species antibodies, HIV-1/2 antibodies and p24 antigen, rubella virus immunoglobulin (Ig) M and IgG, and *Toxoplasma gondii* IgM and IgG in serum, and ethylenediaminetetraacetic acid (EDTA) blood Polymerase Chain Reaction (PCR) for CMV quantitative deoxyribonucleic acid (DNA), human herpesvirus (HHV) type 6 and 8, and *Mycobacterium tuberculosis* (using the tuberculosis-specific interferon gamma release assay (IGRA)). The multidisciplinary team evaluated positive findings and reached consensus about whether the infection was likely to be associated with BGC or should be seen as an incidental finding.

Data reported included age at baseline, age at diagnosis, sex, diagnosis, genetic mutation, Charlson Comorbidity Index (age-adjusted), Total Calcification Score, localization of calcifications, and results of infectious disease diagnostics. Data were presented using mean with standard deviation (SD) for continuous non-skewed variables or median with interquartile range (IQR) for skewed variables, and number with percentage for categorical variables. The sample size was determined based on the number of consecutive patients who visited the outpatient clinic during the study period. A subgroup analysis was performed in patients with versus without a known genetic mutation regarding the prevalence of infectious diseases. Skewed continuous variables were compared using the Mann–Whitney U Test and categorical variables using the chi-squared test. All analyses were performed using IBM SPSS Statistics for Windows, version 27.0 (IBM Corp., Armonk, NY, USA) [11].

Written informed consent was obtained from all participants. Ethical approval was waived by the Dutch Medical Ethical Research Committee NedMec (protocol number 21-170).

### 2.2. Systematic Review

Next, a systematic review was conducted and reported in accordance with the Preferred Reporting Items for Systematic Reviews and Meta-Analyses (PRISMA) guidelines using the ‘PRISMA 2020 Statement’. Completed PRISMA checklists were included in Appendix A. The study protocol was not registered.

A search was carried out using the electronic bibliographic databases MEDLINE and Embase. The search strategy included the term and synonyms of ‘basal ganglia calcifications’. Due to the limited evidence available regarding Fahr’s disease, a broad search term (BGC) was used. The complete search strategy per database was reported in Appendix B. Inclusion criteria were as follows: (1) patients with BGC confirmed by radiological imaging; (2) articles reporting on the etiology or pathophysiology of BGC; (3) articles reporting on at least one infectious disease associated with BGC. Articles were excluded when they concerned the following: (1) articles written in other languages than English or Dutch; (2) case reports; (3) animal studies. No further restrictions were applied regarding the study design. All articles published in the last two decades (from 2002) were searched. Articles published before 2002 were not retrieved due to the large number of articles available. Reference lists of eligible studies were searched for additional references. Unpublished studies were not actively sought.

Screening of titles and abstracts was performed by two reviewers independently and blinded using the prespecified inclusion and exclusion criteria (SB, AG). Disagreement was resolved through discussion between the two reviewers and a third reviewer was consulted if necessary (HK). Full-text screening was performed by two reviewers (SB, BS). The search was rerun prior to final analysis on 1 June 2023 (BS, MP).

The quality of the studies eligible for inclusion was assessed using the Joanna Briggs Institute critical appraisal tools (BS) [12]. The appropriate tool was selected based on study design. A study was considered to be of good quality when only one question was answered with ‘no’ or a maximum of two questions with ‘unclear’; of medium quality when only one question was answered with ‘no’ and a maximum of two with ‘unclear’; and of poor quality when two or more questions were answered with ‘no’ and/or three or more with ‘unclear’. Questions that were answered as ‘not applicable’ were not taken into account when grading the overall quality per eligible study. Studies of medium or poor quality were not excluded due to the limited evidence available for several specific infectious diseases. The overall quality of the included studies was assessed using the Grading of Recommendations, Assessment, Development, and Evaluations (GRADE) tool [13]. The quality of evidence was taken into consideration when developing recommendations for clinical practice.

Data extraction was performed by two reviewers independently (SB and BS) using a prespecified data extraction Excel form. Extracted data included study design, year of publication, country, number of participants, description of study population, age of participants, infectious diseases, and characteristics of brain calcifications identified through radiological imaging, including appearance, size, and localization. Data were presented using descriptive statistics and narrative analyses. Due to the heterogeneity of the included studies, a meta-analysis was not performed.

## 3. Results

### 3.1. Cross-Sectional Study

A total of 59 patients with (suspected) Fahr’s disease or syndrome visited the outpatient clinic during the study period, of whom 4 gave no informed consent for the study and 1 was eventually not diagnosed with Fahr’s disease or syndrome. A total of 54 patients were included in the analysis (flow chart of patient selection included in Appendix C). The median age was 65 years (IQR 47–71) and 44% was male. Most patients were diagnosed with Fahr’s disease (91%). Of the 42 patients who underwent genetic testing, a pathogenic mutation was found in 41% of cases. The most prevalent genetic mutation was identified in the *SLC20A2* gene. The median Total Calcification Score was 30 (range 4 to 66 points). Patient characteristics are presented in Table 1. The clinical characteristics of this specific cohort of patients with Fahr’s disease has been described in a previous paper [9].

Serology tests were negative for *Brucella* sp. and HIV; molecular diagnostics on EDTA-blood revealed no active CMV, HHV6, or HHV8 infection; and IGRA for *Mycobacterium tuberculosis* was negative. Fifteen patients (28%) had a past infection with *Toxoplasma gondii*, and most patients (94%) tested positive for IgG rubella antibodies, either due to prior infection or successful vaccination. Subgroup analyses in patients with versus without a known genetic mutation yielded no significant differences in infectious disease prevalence (past *Toxoplasma gondii* infection in 35% versus 25% of patients, respectively (*p*-value 0.58); positive IgG rubella antibodies in 88% versus 97% of patients, respectively (*p*-value 0.33)) (Appendix D). A complete overview of results of infectious disease testing and localization of intracranial calcifications per patient is presented in Appendix E.

### 3.2. Systematic Review

**Figure 1:**
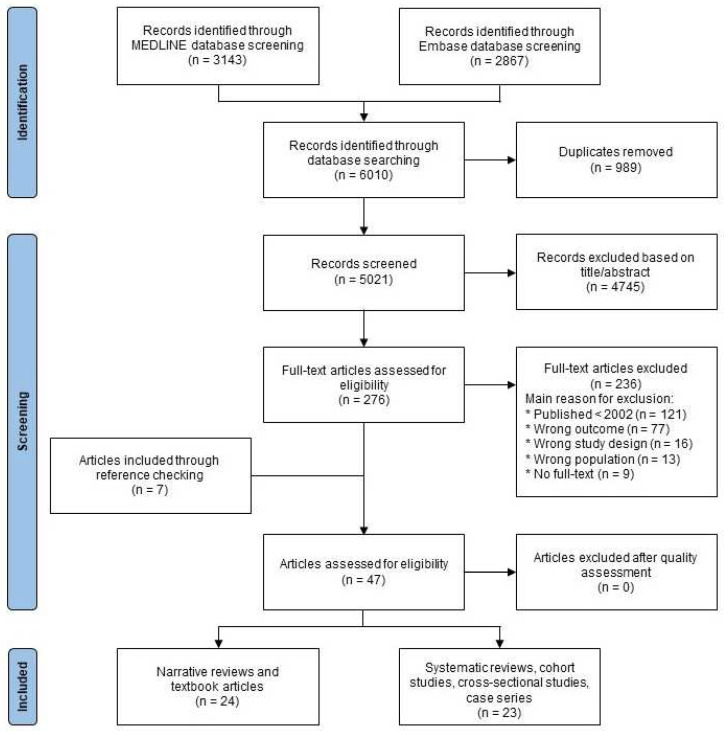
Flow chart of study selection.

The search strategy yielded 5021 unique articles, of which 4745 were excluded after title/abstract screening and 236 after full-text screening (Figure 1). Reference checking of included narrative reviews and textbooks yielded seven additional original studies [14,15,16,17,18,19,20]. In total, 47 articles were included in this systematic review, comprising of 24 narrative reviews or textbook articles and 23 original studies (11 case series, 6 cross-sectional studies, 4 cohort studies and 2 systematic reviews). Characteristics of the included 23 original studies are presented in Table 2. The original studies reported six different pathogens associated with BGC, including CMV, chronic active Epstein–Barr virus (EBV), HIV, *Mycobacterium tuberculosis*, rubella virus, and Zika virus [14,15,16,17,18,19,20,21,22,23,24,25,26,27,28,29,30,31,32,33,34,35,36]. Twenty-one out of the twenty-three original studies regarded congenital or perinatally acquired infectious diseases. The quality of the included original studies was assessed and is presented in Appendix F. The overall quality per study is shown in Table 2. Both systematic reviews were considered to be of poor quality, whilst the quality of the included cross-sectional studies, case series, and cohort studies ranged from poor to good (8 poor, 2 medium, 11 good). The overall quality according to the GRADE tool was considered to be low due to the variable quality and the small number of patients in the studies.

#### 3.2.1. Congenital Infections

##### Cytomegalovirus

CMV disease is the most prevalent congenital viral infection that affects approximately 0.6% of all live births [36,37]. Around 10% of infected newborns are symptomatic [37]. Congenital CMV infections may cause central nervous system malformations, including microcephaly and intracranial calcifications, leading to hearing impairment, visual impairment, and intellectual disability [14,37]. The study of De Vries et al. (2004) described the radiological findings in 11 newborns with symptomatic congenital CMV infections. They found that 10 out of 11 infants had calcifications in the periventricular region and basal ganglia, and/or lenticulostriate vasculopathy [14]. The study of Alarcon et al. (2006) reported 14 newborns with symptomatic CMV infection, of whom 9 had periventricular calcifications and 11 had hyperechogenic areas in the thalamus and basal ganglia [25]. The study of Di Mascio et al. (2023) observed calcifications in the basal ganglia or germinal matrix in 1 out of 10 fetuses on prenatal magnetic resonance imaging (MRI) [36]. No studies were retrieved regarding the development of BGC in adults who acquired a CMV infection later in life.

##### Human Immunodeficiency Virus

HIV is a chronic virus that causes immunodeficiency and may involve several organ systems, including the central nervous system [23]. Congenital and vertically transmitted HIV can affect fetal and infant brain development, causing neurodevelopmental disorders [23,28]. Occurrence of neurological complications of HIV in non-treated children can range from 20 up to 60% [24,28]. BGC are a common finding in HIV-infected children [38]. The cohort study of Tahan et al. demonstrated that, among HIV-infected children, BGC were present in 5 out of 48 cases (10%) [23]. A case series including children with vertically transmitted HIV infections complicated by HIV encephalopathy reported similar findings (BGC present in 4 out of 49 cases (8%)) [28]. The smaller case series of Wilmshurst et al. and Udgirkar et al. reported higher prevalence of BGC of 1 out of 5 (20%) and 2 out of 8 (25%), respectively, in children with HIV encephalopathy [21,24]. Due to the introduction of antiretroviral therapy, HIV has become a chronic disease. The long-term effects of chronic HIV infection and its treatment regimen on the central nervous system are largely unknown. The cohort study of Izbudak et al. demonstrated that in perinatally HIV-infected adolescents with prior stroke, BGC were present in 3 out of 8 cases (38%) [27]. No studies were retrieved regarding the development of BGC in adults who acquired an HIV infection later in life.

##### Rubella Virus

Congenital rubella infections have become rare since the introduction of the rubella vaccine [39,40]. The systematic review of Namiki et al. (2021) reported that in patients with congenital rubella syndrome, parenchymal calcifications were observed in 58%, BGC in 45%, and periventricular calcification in 26% of cases. Other locations for intracranial calcifications included the corpus callosum, deep white matter, and thalamus [35].

##### Zika Virus

Zika virus is mainly transmitted by mosquitoes and has been reported in Africa, the Americas, Asia, and the Pacific. Infection during pregnancy is associated with congenital malformations in newborns [33,41]. Due to the recent outbreak in Brazil in 2015, much research has been published over the past few years [15,16,17,18,19,20,29,30,31,32,33,34,41,42,43,44,45]. Intracranial calcifications are highly prevalent in congenital Zika virus infections. A cross-sectional study published by the Microcephaly Epidemic Research Group in 2016 reported that 85–96% of newborns with microcephaly in Brazil suspected for Zika virus had intracranial calcifications [30]. A systematic review conducted by Radaelli et al. (2020) summarized the neuroimaging findings in newborns with congenital Zika virus: intracranial calcifications were often present in the subcortical area (88–93%), basal ganglia (33–50%), periventricular area (23–37%), brain stem (10–19%), and cerebellum (3–10%) [33]. Other locations where calcifications were reported included the cortical–subcortical junction and thalamus [29,30,31,32,34].

#### 3.2.2. Acquired Infections

##### Epstein–Barr Virus (Chronic Active)

EBV is one of the most common human viruses and is a highly prevalent disease worldwide. Most individuals are infected with EBV at some point in their life. Symptomatology of primary EBV infection varies between asymptomatic to infectious mononucleosis [46]. In rare cases, which are often related to immunodeficiencies, EBV develops a chronic active infection [46]. Chronic active EBV infection is associated with several complications, including central nervous system involvement [26]. The series of 10 cases with chronic active EBV by Ishikawa et al. found that 1 patient had bilateral BGC [26].

##### *Mycobacterium tuberculosis* 

*Mycobacterium tuberculosis* is one of the most common acquired pathogens associated with intracranial calcification [47,48]. Although the lungs are the primary site of infection, approximately 5–10% of infections involve the central nervous system. Intracranial tuberculomas are often observed in high endemic areas [22]. Patients can develop one solitary or multiple tuberculoma lesions, which can be localized in the cerebral hemispheres, basal ganglia, thalamus, cerebellum, and brain stem [22]. These granulomatous lesions may calcify, which may appear as a central nidus of calcification surrounded by a ring of enhancement [48,49]. The study of Wasay et al. reported that in patients with intracranial tuberculoma, 10% of lesions were calcified [22].

#### 3.2.3. Other Infections

Narrative reviews and textbooks reported an additional seven pathogens, which included *Brucella* sp., HSV, mumps virus, *Neisseria meningitidis*, *Taenia solium* (a tapeworm causing cysticercosis), *Toxoplasma gondii*, and *Treponema pallidum* [2,3,4,6,7,17,38,41,43,44,45,47,48,49,50,51,52,53,54,55,56,57,58,59,60]. The original articles, which were cited by the narrative reviews and textbooks, were published before 2002 and were therefore not retrieved. This systematic review found no original studies that reported about one of these seven additional pathogens and was published in or after 2002. An overview of all pathogens associated with BGC in the literature is shown in Appendix G. This overview is based on the original studies and narrative reviews and textbooks that were retrieved by this systematic review and were published between 2002 and June 2023.

## 4. Discussion

We performed an extensive infectious disease diagnostic work-up in non-immunocompromised Dutch patients with Fahr’s disease or syndrome with adult-onset symptoms. During our multi-disciplinary assessment, we found no infections that caused the BGC, although nearly one third had a prior infection with *Toxoplasma gondii* and most patients tested positive for IgG rubella antibodies. Our systematic review demonstrated that the amount and quality of available evidence regarding infectious diseases associated with BGC is limited. During the last two decades, studies have reported associations between BGC and CMV, chronic active EBV, HIV, *Mycobacterium tuberculosis*, rubella virus, and Zika virus [14,15,16,17,18,19,20,21,22,23,24,25,26,27,28,29,30,31,32,33,34,35,36]. Narrative reviews and textbooks have reported associations between BGC and *Brucella* sp., HSV, mumps virus, *Neisseria meningitidis*, *Taenia solium*, *Toxoplasma gondii*, and *Treponema pallidum* [2,3,4,6,7,17,38,41,43,44,45,47,48,49,50,51,52,53,54,55,56,57,58,59,60]. Most original studies reported about congenital or perinatally acquired infectious diseases (CMV, HIV, rubella virus, Zika virus). Studies about acquired pathogens associated with the development of BGC were scarce and included chronic active EBV and *Mycobacterium tuberculosis*. These two studies were performed in non-European countries (Japan and Pakistan, respectively).

The prior *Toxoplasma gondii* infections and positive IgG rubella antibodies in our study population were considered incidental findings with no clinical relevance by the multidisciplinary team. As the rubella vaccine was introduced as part of the Dutch national immunization program in 1974, the high rate of positive IgG rubella antibodies is presumably a combination of both prior infection and successful vaccination in this population [61]. Only congenital, and not later acquired, *Toxoplasma gondii* and rubella virus infections are associated with the development of BGC, and all patients developed symptoms later in life. We found no literature regarding patients with BGC who had a congenital infection that was asymptomatic at birth. Our findings are in agreement with previous research. The seroprevalence of *Toxoplasma gondii* and rubella virus in the general population in the Netherlands is estimated to be 30% and 95%, respectively [62,63]. These similar seroprevalences substantiate the presumption that the prior infections with *Toxoplasma gondii* or positive rubella IgG antibodies are unlikely to be associated with BGC in our study population since the extensive BGC as seen in patients with Fahr’s disease or syndrome is rarely seen in the general population. In addition, the seroprevalences did not differ significantly between patients with and without a known genetic mutation in our patient population, which further affirms our hypothesis.

The diagnostic criteria for Fahr’s disease were developed in 1971 and were last updated in 2005 by Manyam [3,7,64]. However, the first genetic mutation was not discovered until 2012 [65]. Researchers assume not all genetic mutations associated with Fahr’s disease have been identified yet [65]. The recommendations from the previous literature to exclude infectious diseases as underlying causes of BGC were developed in a period when the genetic foundation for BGC was not as solid as it is in present times. Furthermore, the incidences of infectious diseases are changing over time. For example, the introduction of the rubella vaccine drastically reduced the number of cases of congenital rubella [66]. BGC associated with HIV infection are rarely encountered in the Western world nowadays due to antiretroviral therapy [57]. This incidence decline is reflected by the limited number of studies that have been published in the last two decades regarding these diseases. However, the recent Zika virus outbreak in Brazil in 2015 has demonstrated that new introductions of infectious diseases in humans are still occurring. These recent genetic and infectious disease developments emphasize the need for updated recommendations for the diagnostic assessment of patients with BGC, including the infectious disease work-up.

**Figure 2:**
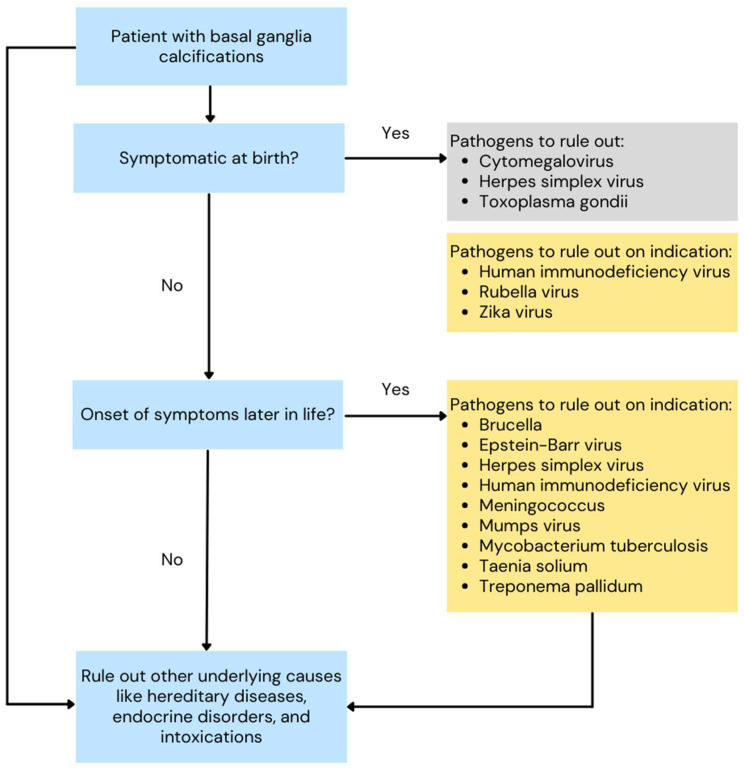
Flow chart for the assessment of infectious diseases in patients with basal ganglia calcifications. Pathogens in grey boxes should always be ruled out. Pathogens in yellow boxes should be ruled out on indication.

Based on the findings of this cross-sectional study and systematic review, we propose recommendations for the assessment of infectious diseases in patients with BGC in Western European countries (Figure 2). We recommend that infectious disease diagnostics should not be routinely performed in all patients with BGC, but that infections should only be ruled out on indication. Examples of indications include patients who were symptomatic at birth; patients who have been in endemic areas; immunocompromised patients; unvaccinated patients or unvaccinated mothers at the time of pregnancy; high-risk behavior; presence of characteristic radiological features (for example intracranial tuberculomas in tuberculosis); and clinical symptoms consistent with the infectious disease. Infectious disease work-up should be omitted in patients with BGC in whom there is no indication to suspect the infectious disease exists. The diagnostic work-up should rather focus on identifying a genetic mutation and excluding other secondary causes like endocrine disorders. These tests should be run concurrently in patients in whom a personalized infectious work-up is performed on indication in order to avoid diagnostic and treatment delay. This approach contributes to a more efficient diagnostic work-up and reduces unnecessary testing and costs.

This is the first study to report the results of an extensive infectious disease work-up in a relatively large population of patients with BGC who were diagnosed with Fahr’s disease or syndrome. In addition, no other systematic reviews have previously been published that summarize the available evidence regarding infectious diseases associated with BGC. A broad search strategy was used with few exclusion criteria in order to limit missing relevant articles. However, our study has several limitations. Our study population consists of patients with Fahr’s disease or syndrome who were diagnosed in adulthood, which introduces selection bias. If, for example, other rare causes of BGC or children were included, this might have affected results. It is known that infectious diseases vary by region and population and change over time. The findings of our cross-sectional study can therefore not be simply generalized onto another population since our study population consists of Dutch and Belgian patients only. Furthermore, in hindsight, our study participants did not have a complete infectious disease diagnostic work-up according to our review. All patients had undergone work-up prior to the conduction of this systematic review. As the infectious disease testing set was formed based on the knowledge we had at that time, not all infections identified through this review were tested and ruled out in our study population. However, based on the findings of our review, it is highly unlikely that we missed any relevant pathogens. This systematic review focusses solely on patients with BGC. Results should not be generalized onto patients with intracranial calcifications without involvement of the basal ganglia. It should be noted that the available evidence retrieved by our search was limited and a relatively large proportion of the included articles was of poor quality (8 out of 23 articles). Articles that were published in other languages than English or Dutch were excluded. It is possible we may have missed articles regarding infections that are only endemic in specific areas if these articles were published in the local language only. Lastly, the review was limited by date and database restrictions. We likely have missed some articles because of these restrictions. This was handled by reference checking, which led to a broad review of infectious diseases.

To conclude, we propose evidence-based recommendations for the assessment of infectious diseases in the diagnostic work-up in patients who present with BGC and adult-onset symptoms in Western countries. Infectious disease diagnostics should not be routinely performed in all patients with BGC, but only on specific indications. The diagnostic work-up should rather focus on identifying a genetic mutation and excluding other secondary causes. This new approach may improve the diagnostic trajectory for patients with BGC by reducing unnecessary testing and costs.

## Figures and Tables

**Table 1:** Patient characteristics of patients with Fahr’s disease or syndrome.

Characteristic	***n*** = 54
Age at baseline	65 (47–71)
Male	24 (44%)
Diagnosis	
Fahr’s disease	49 (91%)
Fahr’s syndrome	5 (9%)
Genetic testing	42 (78%) ^a^
No genetic mutation	20 (48%) ^b^
Results not known yet	5 (12%) ^b^
Known genetic mutation	17 (41%) ^b^
*SLC20A2*	10 (59%) ^c^
*XPR1*	3 (18%) ^c^
*PDGFB*	2 (12%) ^c^
*MYORG*	2 (12%) ^c^
*PDGFRB*	0 (0%) ^c^
*JAM2*	0 (0%) ^c^
Charlson Comorbidity Index (age-adjusted)	2 (1–4)
Total Calcification Score	30 (13–45)
Infectious disease testing	
*Brucella* sp. (*n* = 38)	0 (0%) ^d^
Cytomegalovirus (*n* = 51)	0 (0%) ^d^
Human immunodeficiency virus (*n* = 53)	0 (0%) ^d^
Human herpesvirus type 6 (*n* = 49)	0 (0%) ^d^
Human herpesvirus type 8 (*n* = 50)	0 (0%) ^d^
*Mycobacterium tuberculosis* (*n* = 52)	0 (0%) ^d^
Rubella virus (*n* = 53)	
IgM	0 (0%) ^d^
IgG	50 (94%) ^d^
*Toxoplasma gondii* (*n* = 53)	
IgM	0 (0%) ^d^
IgG	15 (28%) ^d^

Abbreviations: *SLC20A2* = Solute Carrier Family 20 Member 2, *XPR1* = Xenotropic And Polytropic Retrovirus Receptor 1, *PDGFB* = Platelet Derived Growth Factor Subunit B, *MYORG* = Myogenesis Regulating Glycosidase, *PDGFRB* = Platelet Derived Growth Factor Receptor Beta, *JAM2* = Junctional Adhesion Molecule 2, Ig = immunoglobulin. Data were presented as number (percentage) or median (interquartile range). ^a^ As a percentage of the total population. ^b^ As a percentage of the patients who underwent genetic testing. ^c^ As a percentage of the patients with a genetic mutation. ^d^ As a percentage of the patients who underwent testing.

**Table 2:** Characteristics of original studies included in the systematic review.

First Author (Year)	Study Design	Study Population	Pathogen	Localization and Characteristics of Brain Calcifications	Overall Quality
Udgirkar (2003) [21]	Case series	8 children with HIV encephalopathy (aged 1–10 years), in India	HIV	Basal ganglia	Poor
Wasay (2003) [22]	Cross-sectional	100 patients with intracranial tuberculoma (aged 1–75 years), in Pakistan	*Mycobacterium tuberculosis*	In tuberculous granuloma, which can be localized in the basal ganglia	Poor
De Vries (2004) [14]	Case series	11 newborns with congenital CMV, in the Netherlands	CMV	Basal ganglia, periventricular	Good
Tahan (2006) [23]	Cohort	88 children with HIV and 84 children exposed to HIV, but who tested negative, in Brazil	HIV	Basal ganglia	Poor
Wilmshurst (2006) [24]	Case series	7 children with HIV and neurologic manifestations (aged 7 months-6 years), in South Africa	HIV	Basal ganglia	Poor
Alarcon (2006) [25]	Cross-sectional	14 newborns with symptomatic CMV infection, in Spain	CMV	Basal ganglia, cerebellum, cortex, periventricular, thalamus, white matter	Good
Ishikawa (2013) [26]	Case series	14 children with chronic active EBV (aged 1–12 years), in Japan	EBV (chronic active)	Basal ganglia	Good
Izbudak (2013) [27]	Cohort	8 children with perinatally acquired HIV and acute stroke (mean age 18.5 years), in USA	HIV	Basal ganglia, thalamus	Good
Donald (2015) [28]	Case series	87 children with HIV encephalopathy (median age 64 months (IQR 27–95)), in South Africa	HIV	Basal ganglia	Good
Aragao (2016) [15]	Case series	23 newborns with microcephaly and presumed Zika virus-related congenital infection, in Brazil	Zika virus	Basal ganglia, brain stem, cerebellum, periventricular, subcortical–cortical junction (punctate, linear, or coarse)	Good
Cavalheiro (2016) [29]	Cross-sectional	13 newborns with microcephaly born to mothers who were infected by the Zika virus in the early stage of pregnancy, in Brazil	Zika virus	Basal ganglia, periventricular, subcortical–cortical junction (coarse)	Poor
Hazin (2016) [16]	Case series	23 infants with congenital microcephaly who were suspected to have congenital Zika virus infection (mean age 36 days, range 3 days-5 months), in Brazil	Zika virus	Basal ganglia, subcortical–cortical junction, thalamus (punctate, bandlike distribution)	Poor
Melo (2016) [17]	Case series	11 newborns with congenital Zika virus infection, in Brazil	Zika virus	Basal ganglia, brain stem, cerebellum, periventricular, subcortex, thalamus	Good
Microcephaly Epidemic Research Group (2016) [30]	Cross-sectional	104 infants with microcephaly, in Brazil	Zika virus	Basal ganglia, cerebellum, midbrain, periventricular, subcortical–cortical junction, thalamus	Good
Soares de Oliveira-Szejnfeld (2016) [31]	Cross-sectional	45 fetuses/newborns with presumed Zika virus infection with intracranial calcifications, in Brazil	Zika virus	Basal ganglia, brain stem, cerebellum, cortex, periventricular, subcortical–cortical junction, thalamus	Good
Campo (2017) [18]	Cross-sectional	83 infants with microcephaly and presumed Zika virus congenital infection (range 0–10 months), in Brazil	Zika virus	Basal ganglia, periventricular, subcortical–cortical junction	Poor
Castro (2017) [32]	Case series	8 newborns with microcephaly, in Brazil	Zika virus	Basal ganglia, brain stem, periventricular, subcortical–cortical junction	Good
Chimelli (2017) [19]	Case series	10 stillborns/newborns who died within the first 37 h of life with congenital Zika virus infection, in Brazil	Zika virus	Basal ganglia, brain stem, subcortical–cortical junction, thalamus	Poor
Schaub (2017) [20]	Case series	14 fetuses of pregnant women with confirmed Zika virus infection, in Martinique	Zika virus	Basal ganglia, subcortical–cortical junction, thalamus	Medium
Radaelli (2020) [33]	Systematic review	Children with microcephaly due to Zika virus	Zika virus	Basal ganglia, brain stem, cerebellum, periventricular, white matter	Poor
Van der Linden (2020) [34]	Cohort	21 children with congenital Zika virus syndrome (age 16–30 months), in Brazil	Zika virus	Basal ganglia, subcortical–cortical junction	Good
Namiki (2022) [35]	Systematic review	31 infants with congenital rubella virus syndrome (mean age 10.9 months (SD ± 14.7))	Rubella virus	Basal ganglia, corpus callosum, parenchyma, periventricular, thalamus, white matter	Poor
Di Mascio (2023) [36]	Cohort	95 newborns with congenital CMV infection (mean age 26.0 weeks (SD ± 5.1)), in Italy	CMV	Basal ganglia	Medium

Abbreviations: HIV = human immunodeficiency virus, CMV = cytomegalovirus, EBV = Epstein–Barr virus, USA = United States of America, IQR = interquartile range, SD = standard deviation.

**Figure A1:**
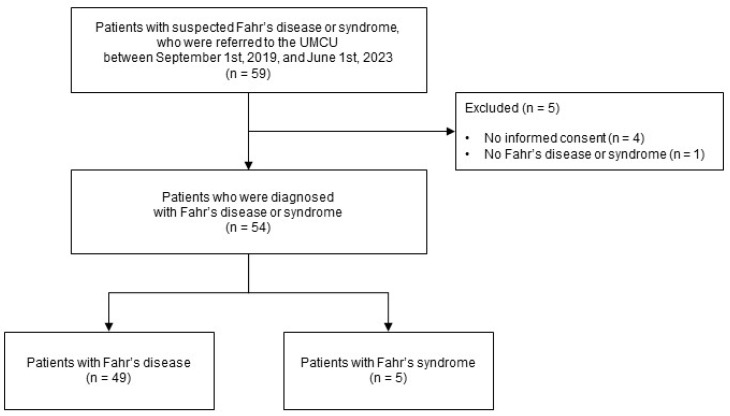
Flow chart of patients included in cross-sectional study. Abbreviations: UMCU = University Medical Center Utrecht.

**Figure A2:**
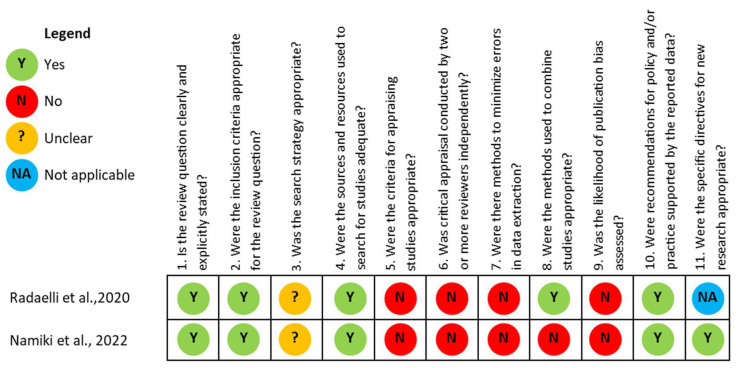
Quality assessment of included systematic reviews [33,35].

**Figure A3:**
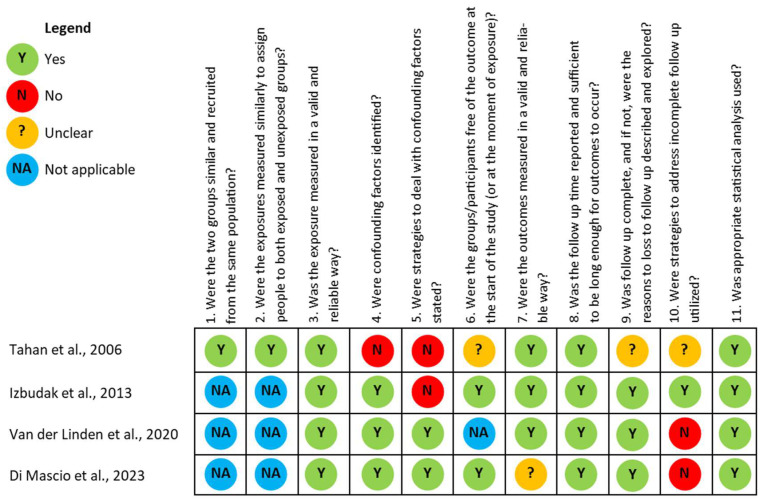
Quality assessment of included cohort studies [23,27,34,36].

**Figure A4:**
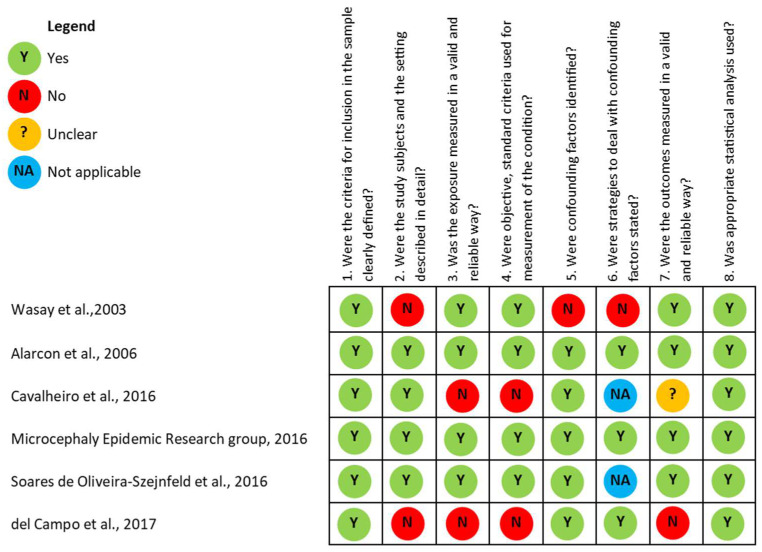
Quality assessment of included cross-sectional studies [18,22,25,29,30,31].

**Figure A5:**
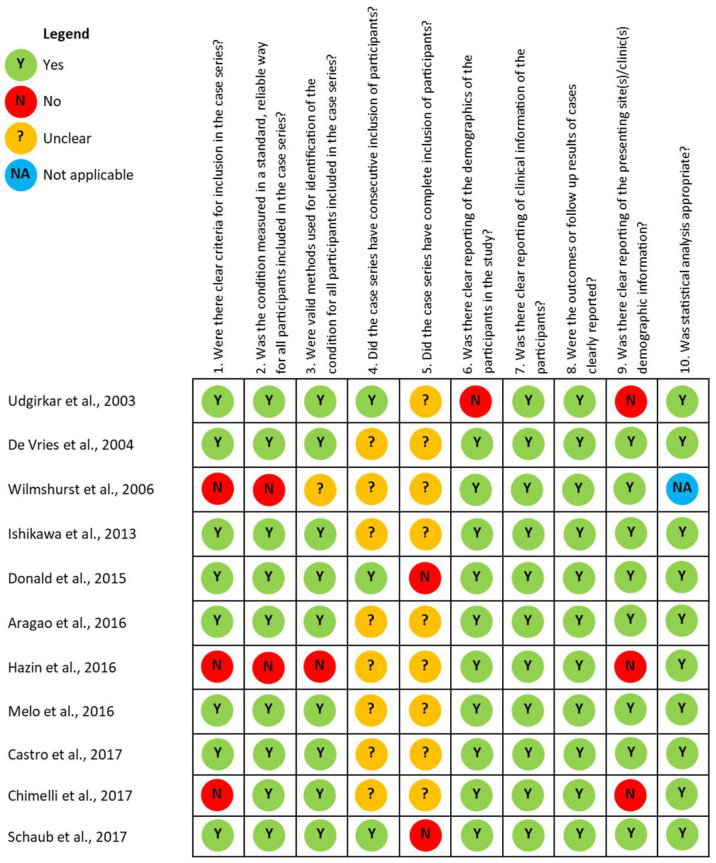
Quality assessment of included case series [14,15,16,17,19,20,21,24,26,28,32].

## Data Availability

The data presented in this study are available on request from the corresponding author.

## Appendix A

**Table A1:** PRISMA checklist [67].

Section and Topic	Item	Checklist Item	Location Where Item Is Reported
**Title**			
Title	1	Identify the report as a systematic review.	Title
**Abstract**			
Abstract	2	See the PRISMA 2020 for Abstracts checklist.	Appendix A, Table A2.
**Introduction**			
Rationale	3	Describe the rationale for the review in the context of existing knowledge.	Section 1, paragraph 1–2
Objectives	4	Provide an explicit statement of the objective(s) or question(s) the review addresses.	Section 1, paragraph 3
**Methods**			
Eligibility criteria	5	Specify the inclusion and exclusion criteria for the review and how studies were grouped for the syntheses.	Section 2.2, paragraph 2
Information sources	6	Specify all databases, registers, websites, organisations, reference lists, and other sources searched or consulted to identify studies. Specify the date when each source was last searched or consulted.	Section 2.2, paragraph 2–3
Search strategy	7	Present the full search strategies for all databases, registers, and websites, including any filters and limits used.	Appendix B
Selection process	8	Specify the methods used to decide whether a study met the inclusion criteria of the review, including how many reviewers screened each record and each report retrieved, whether they worked independently, and if applicable, details of automation tools used in the process.	Section 2.2, paragraph 3
Data collection process	9	Specify the methods used to collect data from reports, including how many reviewers collected data from each report, whether they worked independently, any processes for obtaining or confirming data from study investigators, and if applicable, details of automation tools used in the process.	Section 2.2, paragraph 5
Data items	10a	List and define all outcomes for which data were sought. Specify whether all results that were compatible with each outcome domain in each study were sought (e.g., for all measures, time points, analyses), and if not, the methods used to decide which results to collect.	Section 2.2, paragraph 5
	10b	List and define all other variables for which data were sought (e.g., participant and intervention characteristics, funding sources). Describe any assumptions made about any missing or unclear information.	Section 2.2, paragraph 5
Study risk of bias assessment	11	Specify the methods used to assess risk of bias in the included studies, including details of the tool(s) used, how many reviewers assessed each study and whether they worked independently, and if applicable, details of automation tools used in the process.	Section 2.2, paragraph 4
Effect measures	12	Specify for each outcome the effect measure(s) (e.g., risk ratio, mean difference) used in the synthesis or presentation of results.	Section 2.2, paragraph 5
Synthesis methods	13a	Describe the processes used to decide which studies were eligible for each synthesis (e.g., tabulating the study intervention characteristics and comparing against the planned groups for each synthesis (item #5)).	Section 2.2, paragraph 4
	13b	Describe any methods required to prepare the data for presentation or synthesis, such as handling of missing summary statistics, or data conversions.	Section 2.2, paragraph 4–5
	13c	Describe any methods used to tabulate or visually display results of individual studies and syntheses.	Section 2.2, paragraph 4–5
	13d	Describe any methods used to synthesize results and provide a rationale for the choice(s). If meta-analysis was performed, describe the model(s), method(s) to identify the presence and extent of statistical heterogeneity, and software package(s) used.	Section 2.2, paragraph 5
	13e	Describe any methods used to explore possible causes of heterogeneity among study results (e.g., subgroup analysis, meta-regression).	Not applicable
	13f	Describe any sensitivity analyses conducted to assess robustness of the synthesized results.	Not applicable
Reporting bias assessment	14	Describe any methods used to assess risk of bias due to missing results in a synthesis (arising from reporting biases).	Not applicable
Certainty assessment	15	Describe any methods used to assess certainty (or confidence) in the body of evidence for an outcome.	Section 2.2, paragraph 4
**Results**			
Study selection	16a	Describe the results of the search and selection process, from the number of records identified in the search to the number of studies included in the review, ideally using a flow diagram.	Figure 1
	16b	Cite studies that might appear to meet the inclusion criteria, but which were excluded, and explain why they were excluded.	Not applicable
Study characteristics	17	Cite each included study and present its characteristics.	Table 1, Appendix G
Risk of bias in studies	18	Present assessments of risk of bias for each included study.	Appendix F
Results of individual studies	19	For all outcomes, present for each study (a) summary statistics for each group (where appropriate) and (b) an effect estimate and its precision (e.g., confidence/credible interval), ideally using structured tables or plots.	Table 2
Results of syntheses	20a	For each synthesis, briefly summarise the characteristics and risk of bias among contributing studies.	Table 2, Appendix F
	20b	Present results of all statistical syntheses conducted. If meta-analysis was conducted, present for each the summary estimate and its precision (e.g., confidence/credible interval) and measures of statistical heterogeneity. If comparing groups, describe the direction of the effect.	Section 3.2
	20c	Present results of all investigations of possible causes of heterogeneity among study results.	Not applicable
	20d	Present results of all sensitivity analyses conducted to assess the robustness of the synthesized results.	Not applicable
Reporting biases	21	Present assessments of risk of bias due to missing results (arising from reporting biases) for each synthesis assessed.	Not applicable
Certainty of evidence	22	Present assessments of certainty (or confidence) in the body of evidence for each outcome assessed.	Section 3.2, paragraph 1
**Discussion**			
Discussion	23a	Provide a general interpretation of the results in the context of other evidence.	Section 4, paragraph 1–3
	23b	Discuss any limitations of the evidence included in the review.	Section 4, paragraph 5
	23c	Discuss any limitations of the review processes used.	Section 4, paragraph 5
	23d	Discuss implications of the results for practice, policy, and future research.	Section 4, paragraph 4, 6
**Other InformatioN**			
Registration and protocol	24a	Provide registration information for the review, including register name and registration number, or state that the review was not registered.	Section 2.2, paragraph 1
	24b	Indicate where the review protocol can be accessed, or state that a protocol was not prepared.	Section 2.2, paragraph 1
	24c	Describe and explain any amendments to information provided at registration or in the protocol.	Not applicable
Support	25	Describe sources of financial or non-financial support for the review, and the role of the funders or sponsors in the review.	Conflicts of Interest, Funding
Competing interests	26	Declare any competing interests of review authors.	Conflicts of Interest, Funding
Availability of data, code and other materials	27	Report which of the following are publicly available and where they can be found: template data collection forms; data extracted from included studies; data used for all analyses; analytic code; any other materials used in the review.	Not applicable

**Table A2:** PRISMA 2020 for Abstracts Checklist.

Section and Topic	Item	Checklist Item	Reported (Yes/No)
**Title**			
Title	1	Identify the report as a systematic review.	Yes
**Background**			
Objectives	2	Provide an explicit statement of the main objective(s) or question(s) the review addresses.	Yes
**Methods**			
Eligibility criteria	3	Specify the inclusion and exclusion criteria for the review.	No
Information sources	4	Specify the information sources (e.g., databases, registers) used to identify studies and the date when each was last searched.	Yes
Risk of bias	5	Specify the methods used to assess risk of bias in the included studies.	No
Synthesis of results	6	Specify the methods used to present and synthesise results.	No
**Results**			
Included studies	7	Give the total number of included studies and participants and summarise relevant characteristics of studies.	Yes, expect that the total number of participants was not stated
Synthesis of results	8	Present results for main outcomes, preferably indicating the number of included studies and participants for each. If meta-analysis was conducted, report the summary estimate and confidence/credible interval. If comparing groups, indicate the direction of the effect (i.e., which group is favoured).	Yes
**Discussion**			
Limitations of evidence	9	Provide a brief summary of the limitations of the evidence included in the review (e.g., study risk of bias, inconsistency, and imprecision).	Yes
Interpretation	10	Provide a general interpretation of the results and important implications.	Yes
**Other**			
Funding	11	Specify the primary source of funding for the review.	N/A
Registration	12	Provide the register name and registration number.	N/A

## Appendix B

Search strategy PubMed database:

(((“basal ganglia” [Title/Abstract] OR “basal nuclei” [Title/Abstract] OR “basal nucleus” [Title/Abstract] OR “basal ganglion” [Title/Abstract] OR “Basal Ganglia” [Mesh] OR (“intracranial” [Title/Abstract]) AND (calcificat*[Title/Abstract] OR “calcinosis” [Title/Abstract] OR “calcium deposit*” [Title/Abstract] OR “Calcification, Physiologic” [Mesh:NoExp] OR “Calcinosis” [Mesh:NoExp]))) OR (“fahr” [Title/Abstract] OR “idiopathic basal ganglia calcification*” [Title/Abstract] OR “ibgc” [Title/Abstract] OR “bilateral striopallidodentate calcinosis” [Title/Abstract] OR “primary familial brain calcification” [Title/Abstract] OR “pfbc” [Title/Abstract]) OR “Fahr’s disease” [Supplementary Concept] OR “idiopathic basal ganglia calcification childhood onset” [Supplementary Concept] OR “basal ganglia calcification idiopathic 2” [Supplementary Concept]) AND dutch[Filter] OR english[Filter]) NOT (“Animals” [Mesh] NOT “Humans” [Mesh])

Search strategy Embase database:

((‘basal ganglion:ab or ti’ OR ‘intracranial’ OR ‘basal nuclei:ab or ti’ OR ‘basal nucleus: ab or ti’ OR ‘basal ganglia:ab or ti’) AND ‘calcification: ab or ti’ OR ‘calcinosis:ab or ti’ OR ‘calcium deposit:ab or ti’ OR ‘calcificat:ab or ti’ OR ‘fahr’ OR ‘idiopathic basal ganglia calcification’/exp OR ‘idiopathic basal ganglia calcification’ OR ‘ibgc’ OR ‘bilateral striopallidodentate calcinosis’ OR ‘primary familial brain calcification’/exp OR ‘primary familial brain calcification’ OR ‘pfbc’ OR ‘idiopathic basal ganglia calcification childhood onset’ OR ‘basal ganglia calcification idiopathic 2’) AND [dutch]/lim OR [english]/lim) NOT animals

## Appendix D

**Table A3:** Patient characteristics of patients with Fahr’s disease or syndrome, subanalysis by with versus without known genetic mutation.

Characteristic	Fahr’s Disease/Syndrome Patients with Known Genetic Mutation (***n*** = 17)	Fahr’s Disease/Syndrome Patients with No Known Genetic Mutation (***n*** = 37)	***p***-Value
Age at baseline	55 (47–66)	66 (49–73)	0.083
Male	10 (59%)	15 (39%)	0.149
Diagnosis of Fahr’s disease	17 (100%)	32 (86%)	0.112
Charlson Comorbidity Index (age-adjusted)	1 (0–3)	3 (1–4)	0.098
Total Calcification Score	38 (16–49)	25 (13–39)	0.171
Infectious disease testing			
Rubella virus IgG	15 (88%)	35 (97%) ^a^ (*n* = 36)	0.328
*Toxoplasma gondii* IgG	6 (35%)	9 (25%) ^a^ (*n* = 36)	0.584

Abbreviations: Ig = immunoglobulin. Data were presented as number (percentage) or median (interquartile range). Statistical differences were compared using the Mann–Whitney U Test for continuous variables and chi-squared test for categorical variables. ^a^ As a percentage of the patients who underwent testing.

## Appendix E

**Table A4:** Overview of infectious disease testing and localization of calcifications per case.

						Infectious Disease Testing					Localization of Calcifications								
Case	Sex	Age at Baseline (Years)	Age at Diagnosis (Years)	Diagnosis	Known Genetic Mutation?	CMV PCR	HHV Type 6 PCR	HHV Type 8 PCR	Rubella IgG	*Toxoplasma* *gondii* IgG	Basal Ganglia	Thalamus	White Matter	Cortex	Cerebellum	Vermis	Mesencephalon	Pons	Medulla
1	F	24	24	Fahr’s disease	No	−	−	−	+	−	+	−	−	−	−	−	−	−	−
2	M	67	67	Fahr’s disease	*MYORG*	−	−	−	+	−	+	+	+	-	+	+	+	+	+
3	M	66	65	Fahr’s disease	No	−	−	−	+	−	+	−	−	−	+	−	−	−	−
4	F	67	67	Fahr’s syndrome, caused by idiopathic hypoparathyroidism	No	−	−	−	+	−	+	+	+	−	+	+	+	−	−
5	F	76	76	Fahr’s syndrome, caused by iatrogenic hypoparathyroidism after strumectomy	Not tested	m	m	m	m	m	+	+	+	+	+	+	+	−	−
6	F	45	43	Fahr’s disease	*SLC20A2*	−	−	−	+	−	+	+	+	−	+	+	−	−	−
7	F	41	41	Fahr’s disease	No	−	−	−	+	−	+	+	−	+	+	−	−	−	−
8	F	68	68	Fahr’s disease	No	−	−	−	+	−	+	−	+	+	+	+	−	−	−
9	F	65	49	Fahr’s disease	No	m	m	m	+	−	+	+	+	+	+	+	−	+	−
10	M	72	71	Fahr’s disease	*SLC20A2*	m	m	m	+	+	+	+	+	−	+	+	−	−	−
11	F	73	72	Fahr’s syndrome, caused by pseudohypoparathyroidism	Not tested	−	−	−	+	−	+	+	+	−	+	−	−	−	−
12	F	42	39	Fahr’s syndrome, caused by primary hypoparathyroidism	No	−	−	−	+	−	+	−	+	−	+	−	−	−	−
13	M	47	47	Fahr’s disease	*SLC20A2*	−	−	−	+	−	+	−	+	−	−	−	−	−	−
14	M	71	71	Fahr’s disease	Not tested	−	−	−	+	+	+	−	+	−	+	+	−	−	−
15	M	65	65	Fahr’s disease	*SLC20A2*	−	−	−	+	+	+	+	+	+	+	+	−	−	−
16	F	80	78	Fahr’s syndrome	No	−	−	−	+	+	+	−	+	−	+	+	−	−	−
17	F	53	53	Fahr’s disease	No	−	−	−	+	−	+	+	−	−	−	+	−	−	−
18	M	38	30	Fahr’s disease	No	−	−	−	+	−	+	+	+	−	+	−	−	−	−
19	M	65	59	Fahr’s disease	*SLC20A2*	−	−	−	+	+	+	+	+	+	+	+	−	−	−
20	F	38	37	Fahr’s disease	*SLC20A2*	−	−	−	+	−	+	+	+	−	−	−	−	−	−
21	M	75	75	Fahr’s disease	No	−	−	−	+	−	+	+	+	+	+	+	+	−	−
22	F	36	35	Fahr’s disease	*MYORG*	−	−	−	+	−	+	+	+	+	+	+	+	−	−
23	M	73	73	Fahr’s disease	No	−	−	−	+	+	+	+	+	−	+	−	−	−	−
24	M	59	59	Fahr’s disease	*SLC20A2*	−	−	−	+	−	+	+	+	+	+	+	+	−	−
25	F	69	57	Fahr’s disease	No	−	−	−	+	−	+	+	+	−	+	−	−	−	−
26	M	66	61	Fahr’s disease	Not tested	−	−	−	+	+	+	+	−	+	+	−	−	−	−
27	F	44	44	Fahr’s disease	No	−	m	−	+	−	+	+	+	−	+	−	−	−	−
28	F	71	71	Fahr’s disease	Not tested	−	−	−	+	−	+	+	+	−	+	+	−	−	−
29	M	58	57	Fahr’s disease	No	−	−	−	+	+	+	+	+	+	+	−	−	−	−
30	F	68	68	Fahr’s disease	Not tested	−	−	−	+	+	+	−	−	−	+	+	−	−	−
31	F	20	20	Fahr’s disease	No	−	−	−	+	−	+	−	−	−	−	−	−	−	−
32	F	75	75	Fahr’s disease	Results not known yet	−	−	−	+	+	+	+	+	+	+	+	+	+	−
33	M	55	55	Fahr’s disease	Results not known yet	−	−	−	+	−	+	+	−	−	+	−	−	−	−
34	F	55	55	Fahr’s disease	*XPR1*	−	−	−	+	−	+	+	+	+	+	+	−	−	−
35	F	57	49	Fahr’s disease	*XPR1*	−	−	−	+	+	+	+	+	−	+	+	−	−	−
36	M	18	18	Fahr’s disease	No	−	−	−	+	−	+	−	−	−	−	−	−	−	−
37	F	65	65	Fahr’s disease	No	−	−	−	+	−	+	−	−	−	−	−	−	−	−
38	M	78	78	Fahr’s disease	Not tested	−	−	−	+	−	+	+	+	+	+	+	−	−	−
39	F	52	38	Fahr’s disease	*SLC20A2*	−	−	−	−	+	+	−	−	−	−	−	−	−	−
40	M	40	38	Fahr’s disease	No	−	−	−	−	−	+	−	−	−	+	−	−	−	−
41	M	47	47	Fahr’s disease	*PDGFB*	−	−	−	+	−	+	+	+	−	+	−	−	−	−
42	M	63	63	Fahr’s disease	Not tested	−	−	−	+	−	+	+	+	−	+	−	−	−	−
43	F	76	76	Fahr’s disease	Results not known yet	−	−	−	+	−	+	−	−	−	−	−	−	−	−
44	F	71	71	Fahr’s disease	*SLC20A2*	−	m	m	+	−	+	+	+	+	+	+	−	−	−
45	F	83	78	Fahr’s disease	Not tested	−	−	−	+	−	+	+	+	−	+	−	−	−	−
46	M	47	47	Fahr’s disease	*PDGFB*	−	−	−	+	−	+	−	−	−	+	−	−	−	−
47	M	69	69	Fahr’s disease	*XPR1*	−	−	−	+	+	+	−	−	−	+	−	−	−	−
48	F	69	63	Fahr’s disease	Not tested	−	−	−	+	−	+	+	+	+	+	+	+	+	+
49	F	60	60	Fahr’s disease	No	−	−	−	+	−	+	−	+	−	+	+	−	−	−
50	M	88	88	Fahr’s disease	Not tested	−	−	−	+	+	+	+	+	−	+	+	−	−	−
51	M	72	72	Fahr’s disease	Not tested	−	−	−	+	+	+	+	+	+	−	−	−	−	−
52	F	41	41	Fahr’s disease	Results not known yet	−	−	−	+	−	+	+	−	+	+	−	−	−	−
53	F	57	46	Fahr’s disease	Results not known yet	−	−	−	+	−	+	+	+	+	+	+	+	+	−
54	M	48	48	Fahr’s disease	*SLC20A2*	−	−	−	−	−	+	−	−	−	−	−	−	−	−

Abbreviations: + = positive, − = negative, m = missing. F = female, M = male, CMV = cytomegalovirus, PCR = Polymerase Chain Reaction, HHV = human herpes virus, Ig = immunoglobulin. Only tests that were positive in at least one case were presented in this table. All tests were negative for human immunodeficiency virus (1 missing), rubella IgM (1 missing), *Toxoplasma gondii* IgM (1 missing), *Mycobacterium tuberculosis* (2 missing) and *Brucella* sp. (cases 1–4, 6, and 8–41 tested negative for *Brucella* sp., the other cases were missing).

## Appendix G

**Table A5:** Overview of pathogens associated with basal ganglia calcifications and other intracranial calcifications, based on systematic reviews, cohort studies, cross-sectional studies, case series, narrative reviews, and textbooks, published between 2002 and June 2023.

Pathogen		Localization and Characteristics of Intracranial Calcifications
Congenital	Cytomegalovirus	Basal ganglia [3,6,14,25,44,50,52,54,55,58], cerebellum [3,25], cortex [25,44,47,55], ependyma [55], parenchyma [48,52,58], periventricular [14,25,44,47,49,50,52,54,55,58], subependymal [47,48,49,55], thalamus [25], white matter [25,44,55]
	Herpes simplex virus	Basal ganglia [3,6,47,49], cerebellum [3,55,58] cortex [47,48,54], parenchyma [54], periventricular [47,54], thalamus [47,54]
	Human immunodeficiency virus	Basal ganglia [2,7,21,23,24,28,38,47,50,51,57,58], cerebellum [47,48], periventricular [48,55], white matter [47,50,58]
	Rubella virus	Basal ganglia [3,6,35,47,48,49,54,55,58], brain stem [48,49,54], cerebellum [3], corpus callosum [35], cortex [47], parenchyma [35], periventricular [35,48,49,54,55,58], thalamus [35]
	*Toxoplasma gondii*	Basal ganglia [2,3,6,44,47,48,49,50,51,54,55,58], cerebellum [3], cortex [44,47,48,49,55,58], meninges [2], parenchyma [2], periventricular [44,47,48,49,54,55,58], subependymal [47], thalamus [44,48,58], ring-enhancing lesions with edema and calcifications [6]. Calcifications may resolve after treatment [47,48,54,58]
	Zika virus	Basal ganglia [15,16,18,19,20,29,30,31,32,33,34,43,44,45], brain stem [15,17,19,31,32,33,44], cerebellum [15,17,30,31,33,43,44,45], cortex [31,44], midbrain [30], periventricular [15,17,18,29,30,31,32,33,44], subcortical–cortical junction [15,16,17,18,19,20,29,30,31,32,33,34,43,44,45,58], thalamus [16,17,19,20,30,31,43,44]
Acquired	*Brucella* sp.	Basal ganglia [2,3,6], cerebellum [2], white matter [2]
	Epstein–Barr virus (chronic active)	Basal ganglia [7,26]
	Herpes simplex virus	Basal ganglia, cortex, thalamus [53]
	Human immunodeficiency virus	Basal ganglia [6,47], parenchyma [2]
	Mumps virus	Basal ganglia [7]
	*Mycobacterium tuberculosis*	In tuberculoma lesions [22,48,50,51,54,56,58], meninges [47,48]. Target sign: central nidus of calcification surrounded by ring of enhancement [47,49,56,58]
	*Neisseria meningitidis*	Basal ganglia [7]
	*Taenia solium*	In dead larva (small, calcified cyst containing an eccentric calcified nodule) in subarachnoid spaces in basal ganglia, convexities, parenchyma (especially subcortical–cortical junction), ventricles [6,47,48,50,51,54,56,58]
	*Treponema pallidum*	Basal ganglia [7]
